# Impella preserves haemodynamics with adequate stressed blood volume and normal pulmonary vascular resistance in a goat model of ventricular fibrillation

**DOI:** 10.1093/ehjopen/oeaf173

**Published:** 2025-12-18

**Authors:** Shohei Yokota, Takaaki Maruhashi, Midori Kakuuchi, Takuya Nishikawa, Hiroki Matsushita, Hidetaka Morita, Kei Sato, Yuki Yoshida, Toru Kawada, Kazunori Uemura, Tomohiro Nishinaka, Keita Saku

**Affiliations:** Department of Cardiovascular Dynamics, National Cerebral and Cardiovascular Center Research Institute, 6-1 Kishibe-Shimmachi, Suita, Osaka 564-8565, Japan; Department of Emergency and Critical Care Medicine, Kitasato University School of Medicine, 1-15-1, Kitazato, Minami-ku, Sagamihara-shi, Kanagawa 252-0373, Japan; Department of Cardiovascular Dynamics, National Cerebral and Cardiovascular Center Research Institute, 6-1 Kishibe-Shimmachi, Suita, Osaka 564-8565, Japan; Department of Research Promotion and Management, National Cerebral and Cardiovascular Center Research Institute, 6-1 Kishibe-Shimmachi, Suita, Osaka 564-8565, Japan; Department of Cardiovascular Dynamics, National Cerebral and Cardiovascular Center Research Institute, 6-1 Kishibe-Shimmachi, Suita, Osaka 564-8565, Japan; Department of Cardiovascular Dynamics, National Cerebral and Cardiovascular Center Research Institute, 6-1 Kishibe-Shimmachi, Suita, Osaka 564-8565, Japan; Department of Cardiovascular Dynamics, National Cerebral and Cardiovascular Center Research Institute, 6-1 Kishibe-Shimmachi, Suita, Osaka 564-8565, Japan; Department of Cardiovascular Dynamics, National Cerebral and Cardiovascular Center Research Institute, 6-1 Kishibe-Shimmachi, Suita, Osaka 564-8565, Japan; Department of Cardiovascular Dynamics, National Cerebral and Cardiovascular Center Research Institute, 6-1 Kishibe-Shimmachi, Suita, Osaka 564-8565, Japan; Department of Cardiovascular Dynamics, National Cerebral and Cardiovascular Center Research Institute, 6-1 Kishibe-Shimmachi, Suita, Osaka 564-8565, Japan; Department of Artificial Organs, National Cerebral and Cardiovascular Center Research Institute, 6-1 Kishibe-Shimmachi, Suita, Osaka 564-8565, Japan; Department of Cardiovascular Dynamics, National Cerebral and Cardiovascular Center Research Institute, 6-1 Kishibe-Shimmachi, Suita, Osaka 564-8565, Japan

**Keywords:** Impella, Ventricular fibrillation, Haemodynamics, Perfusion, Stressed blood volume, Pulmonary vascular resistance

## Abstract

**Aims:**

Ventricular fibrillation (VF) is a frequent and life-threatening complication of acute myocardial infarction. Although the Impella device provides circulatory support solely to the left ventricle (LV), emerging reports have suggested that it may partially maintain haemodynamics even during VF. This study sought to examine this phenomenon under controlled experimental conditions.

**Methods and results:**

In eight Saanen goats, we recorded right atrial pressure (RAP), left atrial pressure (LAP), arterial pressure (AP), LV pressure, pulmonary artery flow (PAF), carotid artery flow (CAF), and coronary flow (CoF). We inserted an Impella CP and induced VF using direct current. In Protocol 1, we changed the stressed blood volume (SBV) by blood withdrawal/infusion or pulmonary vascular resistance (PVR) by pulmonary microembolisation and measured the haemodynamics during VF with Impella P4 support (*n* = 1 each). In Protocol 2 (*n* = 6), we compared the central and peripheral blood flows between the baseline conditions and three Impella support levels (P0, P4, and P8) during VF. In Protocol 2, an increase of Impella support to P4 and P8 during VF significantly increased AP in a support level-dependent manner (P0, P4, and P8: 26.5 ± 2.5, 57.7 ± 8.7 and 72.0 ± 15.3 mmHg, respectively; *P* < 0.001) while lowering RAP and LAP. Impella partially maintained PAF (0, 1.2 ± 0.6 and 1.6 ± 0.9 L/min; *P* = 0.011), CAF (0, 177.6 ± 26.3 and 235.5 ± 44.1 mL/min; *P* < 0.001), and CoF (0, 68.5 ± 32.0 and 57.8 ± 20.6 mL/min; *P* = 0.002) during VF.

**Conclusion:**

Impella preserved systemic, cerebral, and coronary blood flow with adequate SBV and normal PVR conditions in a goat model of VF.

## Graphical Abstract

**Graphical Abstract:**
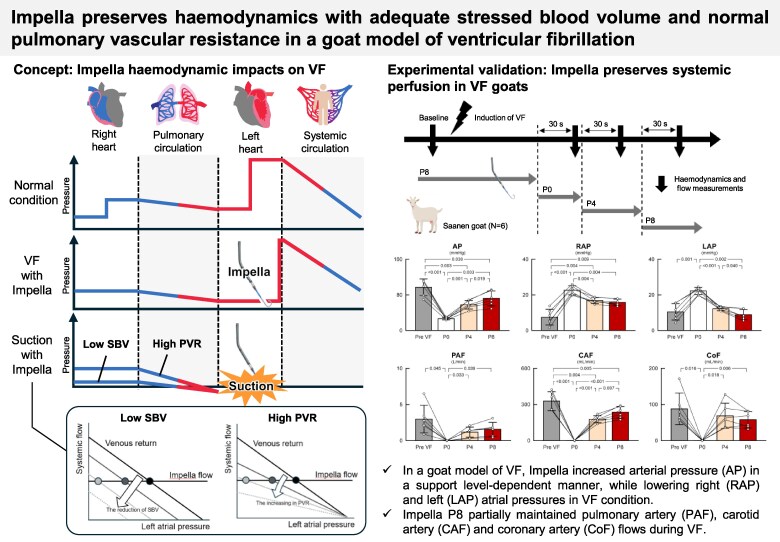

## Introduction

Ventricular fibrillation (VF) following acute myocardial infarction (AMI) is a life-threatening but common clinical problem requiring immediate detection and treatment. Despite advances in reperfusion therapy, VF following AMI remains a significant cause of morbidity and mortality.^1^ While restoring sinus rhythm is the primary goal in VF management, mechanical circulatory support is often used to maintain haemodynamics.^2^

Impella (Abiomed, Danvers, MA, USA) is a catheter-based microaxial flow pump placed across the aortic valve in the left ventricle (LV). It offers rapid haemodynamic support and has been approved worldwide as a device for cardiogenic shock.^3^ Because Impella supports only the LV, its official manual recommends reducing the Impella flow rate during cardiopulmonary resuscitation (CPR) procedure in the event of VF.^4^ However, several cases have been reported in which Impella maintained adequate haemodynamics, even under VF conditions.^5^ Caminiti *et al*.^6^ reported a case of Impella-supported percutaneous coronary intervention (PCI), in which the Impella preserved the consciousness of the patient during VF.

Sufficient LV return is necessary for the stable operation of the Impella. Pulmonary circulation is maintained by a balance between driving pressure, pulmonary artery pressure (PAP), and downstream left atrial pressure (LAP). Under VF conditions, disruption of both left and right ventricular systolic function leads to the absence of pressure gradients between the pulmonary artery and vein, resulting in the collapse of pulmonary circulation. Impella creates a pressure gradient by decreasing LV pressure (LVP) and LAP. Appropriate right atrial pressure (RAP) and pulmonary vascular resistance (PVR) are prerequisites for maintaining pulmonary circulation during VF with Impella support.

This study aimed to elucidate the impact of Impella support on haemodynamics during VF. First, we confirmed the Impella operation during VF by evaluating the effects of stressed blood volume (SBV), a major determinant of venous return, and PVR on Impella-supported circulation during VF in goats. Second, we investigated the amount of central and peripheral circulation under Impella support during VF in goats. Given the exploratory nature of the experimental protocols, this study was designed as a concept-generating investigation.

In addition, this study was intended to physiologically characterize how VF, when it occurs under Impella support, affects circulatory dynamics and Impella flow in an acute experimental setting. It was not designed to propose or advocate Impella-based management strategies for VF, nor should the findings be interpreted as suggesting Impella use for routine VF care.

## Methods

### Animal preparation

Experimental animals were maintained in accordance with the guidelines of the Committee on Animal Studies at the National Cerebral and Cardiovascular Center, and the study was approved by the Animal Investigation Committee (No. 24003). The animals received humane care in compliance with the ‘Principles of Laboratory Animal Care’ formulated by the National Society for Medical Research and the ‘Guide for the Care and Use of Laboratory Animals’ prepared by the Institute of Laboratory Animal Resources and published by the National Institutes of Health (NIH Publication No. 86–23, revised 1996).

We used eight adult Saanen goats (Inoue Shoten, Gunma, Japan). Each animal was sedated with an intramuscular injection of xylazine hydrochloride (1–1.5 mg/kg). Under mechanical ventilation, general anaesthesia was induced and maintained by isoflurane inhalation (1–2 vol%/100 mL in oxygen) and venous injection of rocuronium bromide (0.1–0.15 mg/kg). We measured the systemic arterial pressure (AP) and LVP using a catheter-tipped micromanometer (model PC-751; Millar Instruments, Houston, TX, USA) placed in the ascending aorta and LV via the femoral artery. Additionally, RAP and PAP were measured using a 7.5-Fr pulmonary artery catheter inserted through the jugular vein and positioned in the main trunk of the pulmonary artery. After left thoracotomy in the fourth intercostal space, a pressure line was directly inserted through the left atrial appendage to monitor LAP. Ultrasonic flow metres (Transonic System, Ithaca, NY, USA) were placed on the main pulmonary artery trunk (20 mm), right carotid artery (3.5 mm), and left circumflex coronary artery (2.5–3.5 mm) to measure pulmonary artery flow (PAF), carotid artery flow (CAF), and coronary artery flow (CoF), respectively.

We used the Impella CP® (Abiomed Inc., Danvers, MA, USA), assuming a clinical scenario in which VF occurs under Impella support after acute-phase treatments, such as AMI. An Impella CP catheter was inserted into the LV through a 14-Fr sheath via the left carotid artery. To avoid position-related LV suction as much as possible, we checked the positional relationship between the LV wall and Impella pump inlet using fluoroscopy immediately after insertion and before initiating each protocol. Because goats have ventricular lengths and cardiac geometry that differ from those of humans, the full-length, tip-intact Impella CP did not consistently accommodate the ventricular anatomy, often resulting in suboptimal inlet positioning. Therefore, as previously reported,^7^ we trimmed the distal tip of the Impella catheter to facilitate proper placement in the LV and to enable insertion using a 0.025-inch guidewire, which is thicker than the standard 0.018-inch wire.

Ventricular fibrillation was induced by direct-current stimulation using a 9 V dry battery with wires connected to the positive and negative terminals.^8^ The exposed wire tips were applied to the LV epicardium to induce VF (see Supplementary material online, video  *S1*). After VF induction, residual atrial contractions transiently persisted in some animals, producing distinct atrial contraction waves in the atrial pressure waveform (see Supplementary material  *S1* for details). These atrial contractions gradually disappeared as VF progressed. In two goats in Protocol 2, however, the atrial contractions persisted throughout the entire VF period, and their atrial pressure tracings displayed only minor residual atrial contraction waves. Importantly, all animals in both Protocol 1 and Protocol 2 demonstrated VF, during which effective ventricular contractions were completely abolished.

### Protocols

#### Protocol 1: impact of SBV and PVR on haemodynamics during VF with Impella support (*Figure 1*)

Considering Guyton’s circulatory equilibrium,^9^ SBV and PVR are the major determinants of LV filling and venous return to the heart during VF (see the supplementary material  *S2* for a detailed explanation of Guyton’s circulatory equilibrium during VF). We observed haemodynamics while altering the SBV or PVR in a stepwise manner during VF under Impella support. The level of Impella support was fixed at P4. In Protocol 1-A (*n* = 1), we conducted a stepwise blood withdrawal of 100 mL every 60 s to decrease SBV and continuously observed the haemodynamics. In Protocol 1-B (*n* = 1), we injected 2.0 g of glass microspheres (90 µm in diameter) into the pulmonary artery every 60 s to increase PVR and observed haemodynamics continuously. In Protocols 1 and 2, the procedure was repeated until the LVP was < 0 mmHg.

**Figure 1:**
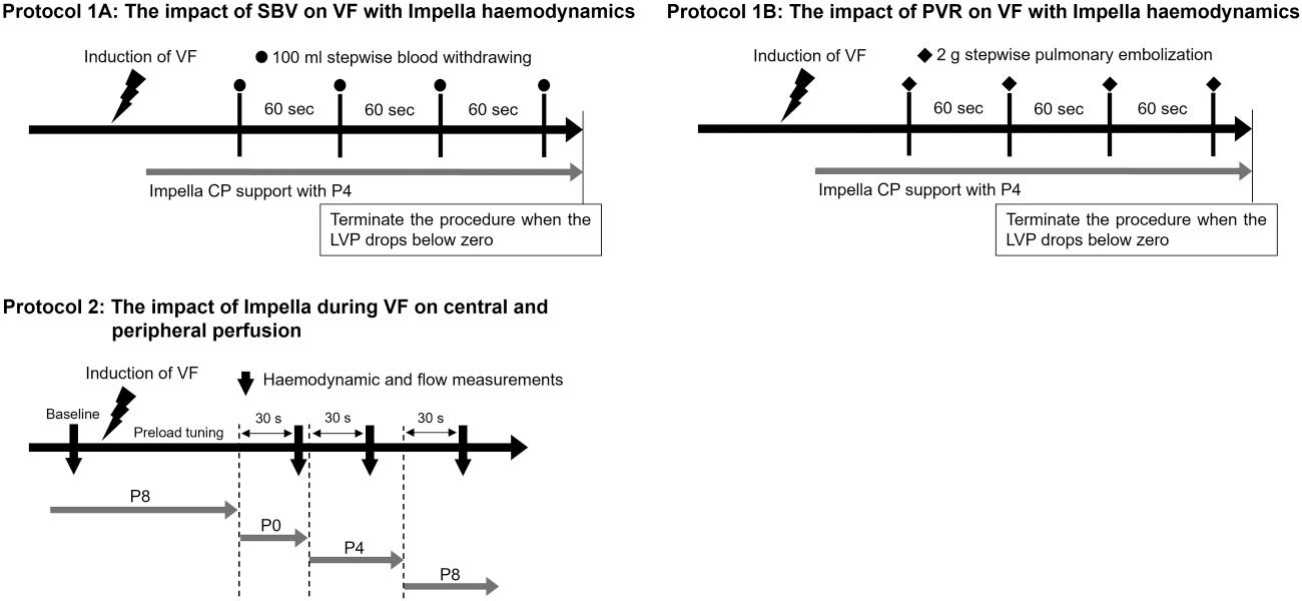
Protocol 1. We confirmed whether the haemodynamic effects of Impella during VF were consistent with the circulatory equilibrium theory in models of bleeding (*A*) and acute pulmonary microembolization (*B*) in protocol 1. SBV, stressed blood volume; VF, ventricular fibrillation; PVR, pulmonary vascular resistance. Protocol 2. We evaluated the impact of Impella support on central and peripheral perfusion during VF using protocol 2.

#### Protocol 2: impact of Impella support on central and peripheral perfusion during VF (*Figure 1*)

We utilized six goats (49.8 ± 3.1 kg) in Protocol 2 to evaluate the impact of Impella on haemodynamics during VF. First, we measured haemodynamics in the sinus rhythm condition supported by Impella at the P8 level as baseline. We then induced VF under Impella support at the P8 level and adjusted SBV by rapid saline infusion to maintain RAP and LVP above 10 mmHg. Following haemodynamic stabilization during VF with Impella P8 support, we successively changed the Impella support level to P0, P4, and P8. As the VF condition with no Impella support (P0 level) renders the animal in critical haemodynamic conditions, the period of the Impella P0 level needed to be shorter (30 s) compared with other levels.

### Data sampling and statistical analysis

We digitized the time-series data at 200 Hz using a 16-bit analogue-to-digital converter (PowerLab 16/35; AD Instruments, USA) and stored them on a dedicated laboratory computer system. Data are presented as mean ± standard deviation. All statistical analyses were performed using EZR in R.^10^ More precisely, it is a modified version of the R commander designed to add statistical functions frequently used in biostatistics.

For the analyses in Protocol 2, to ensure consistency across Impella support levels, haemodynamic measurements were defined as the mean value over a 3-s window beginning 30 s after VF induction or after each Impella flow adjustment. During VF at Impella P0, haemodynamic collapse typically limited safe observation to just over 30 s. The same 30-s timepoint and averaging window were applied uniformly across all Impella settings (P0, P4, and P8) to avoid biased selection of more favourable periods. The differences among the four conditions were examined using repeated-measures one-way analysis of variance (ANOVA) with the Greenhouse–Geisser correction, followed by Tukey tests for all pairwise comparisons. Because measurements were obtained sequentially from the same animals across Impella support levels, repeated-measures ANOVA was used to account for within-animal dependence and avoid assuming independence among observations.

To avoid misinterpretation of zero-offset noise as physiological flow, we set very low flow signals to zero based on known characteristics of transit-time flowmeters. According to manufacturer specifications (Transonic System, Ithaca, NY, USA) and our institutional experience, flows <0.05 L/min for PAF and <0.5 mL/min for coronary/carotid flow CoF and CAF were treated as zero.

We calculated SBV using the previously reported equation as follows.


$$SBV=(CO+G_{S}\cdot RAP+G_{P}\cdot LAP)\cdot W$$

In this study, we set Gp = 3.49, Gs = 19.61, and W = 0.129, as reported by Uemura *et al*.^9^ PVR was calculated using the following equation:


$$PVR=\frac{\left(mean\;PAP-mean\;LAP\right)}{mean\;PAF}$$

Coronary perfusion pressure (CPP) was calculated using the following equation:


CPP=MeanAP−MeanRAP

## Results

### Impact of SBV and PVR on haemodynamics during VF with Impella support

**Figure 2:**
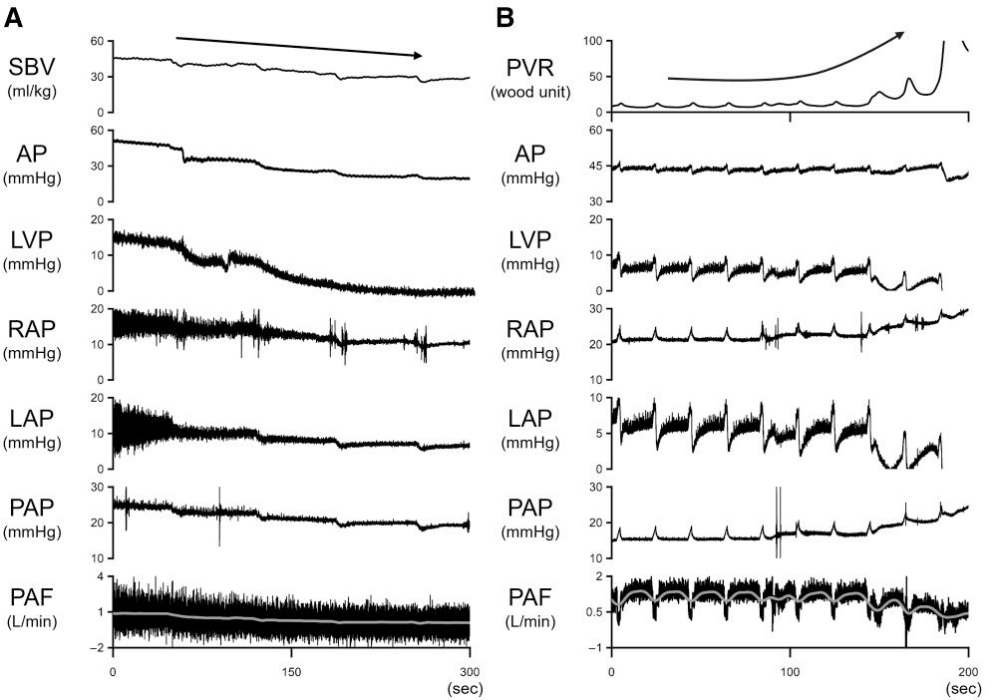
Time series of haemodynamics during VF with Impella support. We observed the haemodynamics during VF with Impella support (P4) under both haemorrhagic (*A*) and stepwise pulmonary hypertension conditions (*B*). We conducted stepwise blood withdrawal of 100 mL every 60 s in protocol 1A and injected 2.0 g of glass microspheres (90 µm in diameter) into the pulmonary artery every 60 s in protocol 1B. As indicated by the arrow, the SBV gradually decreased with blood withdrawal, and the PVR significantly increased after the last injection. SBV, stressed blood volume; AP, arterial pressure; LVP, left ventricular pressure; RAP, right atrial pressure; LAP, left atrial pressure; PAP, pulmonary artery pressure; PAF, pulmonary artery flow; PVR, pulmonary vascular resistance.

*Figure 2-A* shows the haemodynamic changes induced by blood withdrawal. Repeated blood withdrawal (marked by black arrows) gradually decreased the SBV. Corresponding to the SBV decreases, AP, RAP, LAP, and PAP showed a progressive stepwise decline. After the fourth blood withdrawal, PAF decreased to around 0.1 L/min, and LVP fell below zero, indicating the induction of Impella suction.

*Figure 2-B* demonstrates the haemodynamic changes induced by repeated pulmonary embolization. Each injection of microspheres is indicated by an arrow. Although PVR and haemodynamic parameters were stable until the third injection (8 g of microspheres), the fourth injection acutely increased PVR, leading to sudden drops in AP and LVP and induced Impella suction.

### Impact of Impella support on flows and pressures under VF condition

In Protocol 2, three goats required saline infusion before initiating the protocol. After stabilization, RAP was 16.1 ± 2.3 mmHg under Impella P8 support.

**Figure 3:**
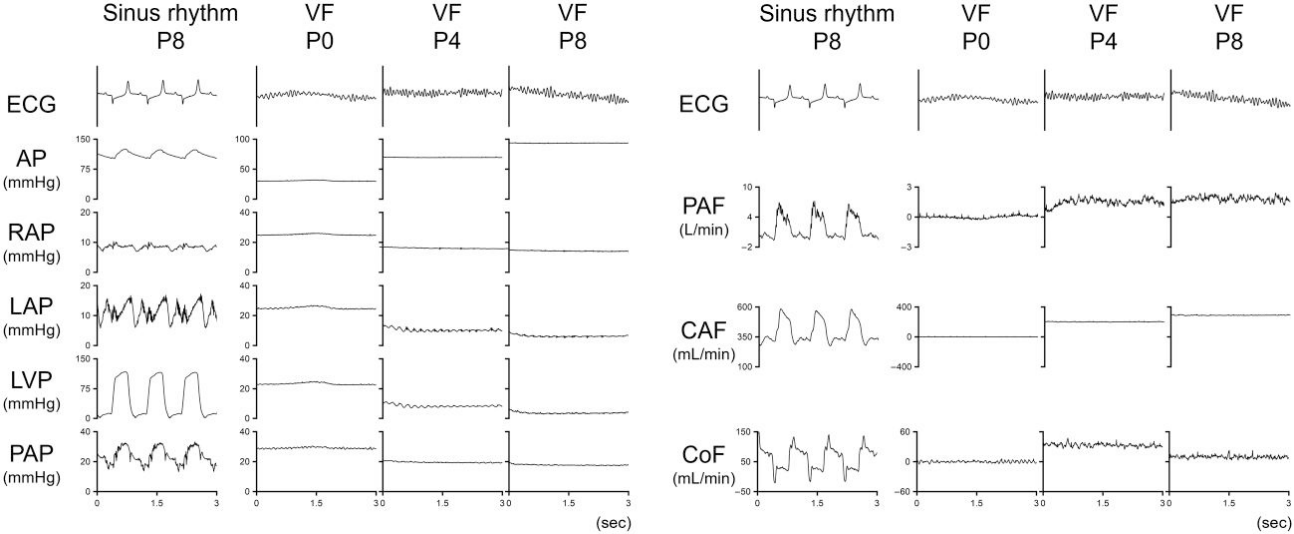
Time series of haemodynamics during VF with several support levels of Impella. Representative waveforms of pressure and flow changes during VF at three Impella support levels (P0, P4, and P8). A stepwise increase in Impella support levels from P0 to P8 increased AP, PAF, CAF, and CoF while reducing RAP, LAP, and LVP. ECG, electrocardiogram; AP, arterial pressure; RAP, right atrial pressure; LAP, left atrial pressure; LVP, left ventricular pressure; PAP, pulmonary artery pressure; PAF, pulmonary artery flow; CAF, carotid artery flow; CoF, coronary artery flow.

**Figure 4:**
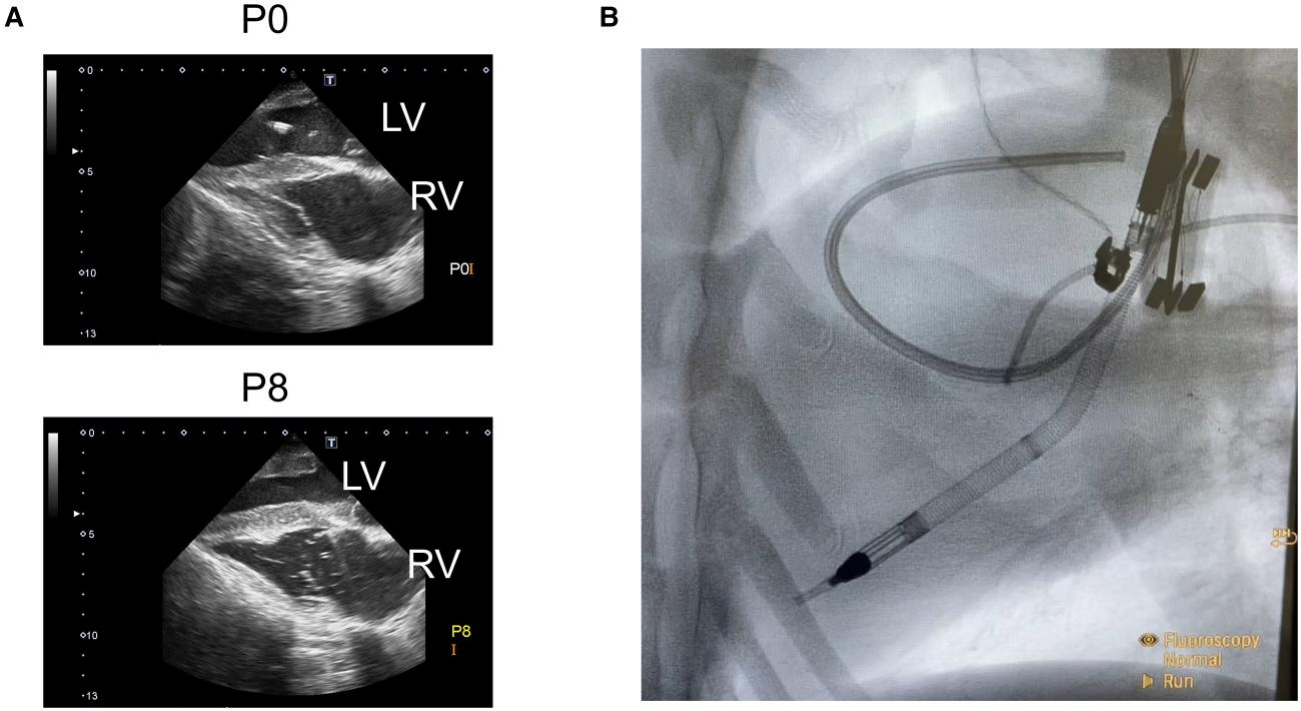
Echocardiogram and fluoroscopic view during VF with Impella support. We attached the echo probe directly to the LV and assessed the echocardiogram of one goat. An increase in the Impella support level reduced the LV chamber size. We also confirmed the Impella position using fluoroscopy. LV, left ventricle; RV, right ventricle.

*Figure 3* shows the representative waveforms of the pressure and flow changes during VF under the three levels of Impella support. A stepwise increase in Impella support levels from P0 to P8 increased AP, PAF, CAF, and CoF while reducing RAP, LAP, and LVP. As shown in *Figure 4-A*, Impella P8 reduced LV size compared to no Impella support (P0) during VF. We also confirmed the Impella position using fluoroscopy (*Figure 4-B*).

**Figure 5:**
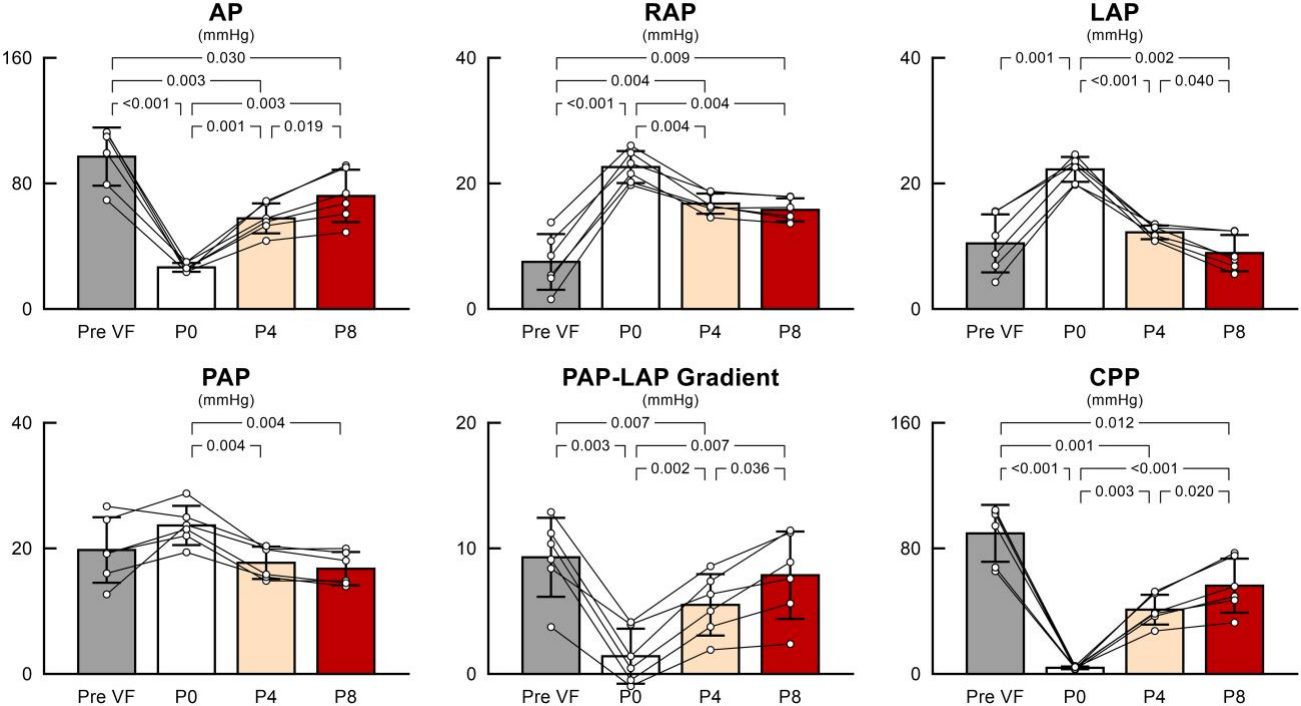
The impact of Impella on systemic, pulmonary, and atrial pressures during VF. Each bar shows the mean value together with individual data (*n* = 6). Differences between the four conditions were examined using repeated-measures one-way analysis of variance with Greenhouse–Geisser correction, followed by Tukey’s test for all pairwise comparisons. VF, ventricular fibrillation; AP, arterial pressure; RAP, right atrial pressure; LAP, left atrial pressure; PAP, pulmonary artery pressure; CPP, coronary perfusion pressure.

**Figure 6:**
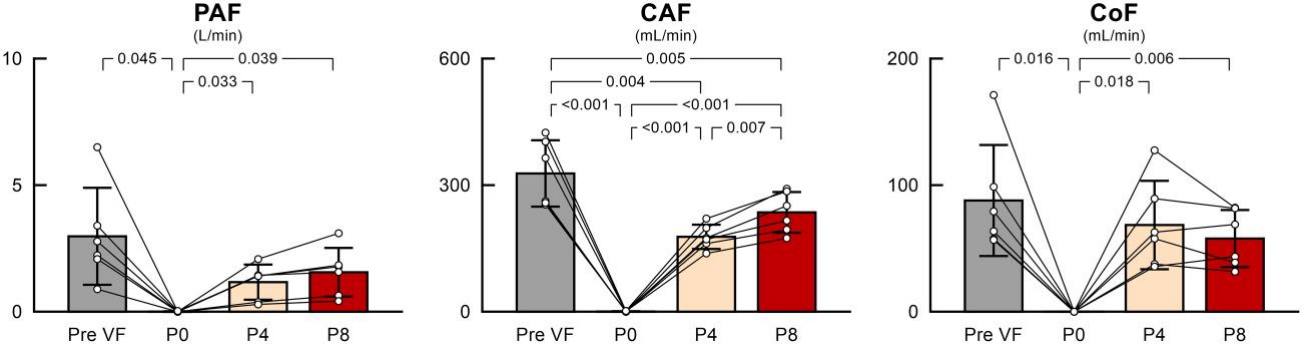
The impact of Impella on systemic, carotid, and coronary flows during VF. Each bar shows the mean value together with individual data (*n* = 6). Differences between the four conditions were examined using repeated-measures one-way analysis of variance with Greenhouse–Geisser correction, followed by Tukey’s test for all pairwise comparisons. VF, ventricular fibrillation; PAF, pulmonary artery flow; CAF, carotid artery flow; CoF, coronary artery flow.

*Figure 5* summarizes the changes in systemic, pulmonary, and atrial pressures during VF at various levels of Impella support. In goats initially in sinus rhythm supported by Impella P8 support (baseline), changing the Impella support level to P0 under VF conditions and increasing the support level to P4 and P8 during VF significantly increased AP in a support level-dependent manner (baseline, P0, P4, and P8: 97.1 ± 16.9, 26.5 ± 2.5, 57.7 ± 8.7, and 72.0 ± 15.3 mmHg, respectively; *P* < 0.001) while lowering RAP (7.5 ± 4.1, 22.6 ± 2.3, 16.8 ± 1.5, and 15.8 ± 1.7 mm Hg; *P* < 0.001), LAP (10.5 ± 4.2, 22.2 ± 1.8, 12.2 ± 1.0, and 8.9 ± 2.6 mmHg; *P* < 0.001), and PAP (19.7 ± 4.8, 23.7 ± 2.9, 17.7 ± 2.4, and 16.8 ± 2.4 mmHg; *P* = 0.022), and augmenting CPP (89.6 ± 16.5, 3.9 ± 0.8, 41.0 ± 8.7, and 56.3 ± 15.7 mmHg; *P* < 0.001). The PAP–LAP gradient, the driving pressure for pulmonary blood flow, fell to zero in VF with Impella P0 and was significantly increased by Impella P4 and P8 supports (9.3 ± 2.9, 1.4 ± 2.0, 5.5 ± 2.2, and 7.9 ± 3.2 mmHg; *P* < 0.001). As summarized in *Figure 6*, Impella maintained PAF (baseline, P0, P4, and P8: 3.0 ± 1.8, 0, 1.2 ± 0.6, and 1.6 ± 0.9 L/min, respectively; *P* = 0.011), CAF (327.8 ± 71.9, 0, 177.6 ± 26.3, and 235.5 ± 44.1 mL/min, respectively; *P* < 0.001), and CoF (87.8 ± 40.4, 0, 68.5 ± 32.0, and 57.8 ± 20.6 mL/min; *P* = 0.002) during VF. Impella P8 support during VF maintained 55.7 ± 20.8% of PAF, 72.5 ± 5.6% of CAF, and 68.8 ± 17.4% of CoF compared to the values at baseline.

## Discussion

The major findings of this study are as follows: (i) a decrease in SBV and an increase in PVR reduced LV filling and attenuated the stable operation of the Impella during VF. (ii) During VF, the maximum support level (P8) of Impella preserved systemic, coronary, and cerebral blood flow and demonstrated partial restoration of haemodynamics.

### Haemodynamic mechanism of Impella during VF

As the appropriate haemodynamic operating point is determined by the venous return curve, we investigated how changes in SBV and PVR affect the operating point of haemodynamics during VF with Impella support.

**Figure 7:**
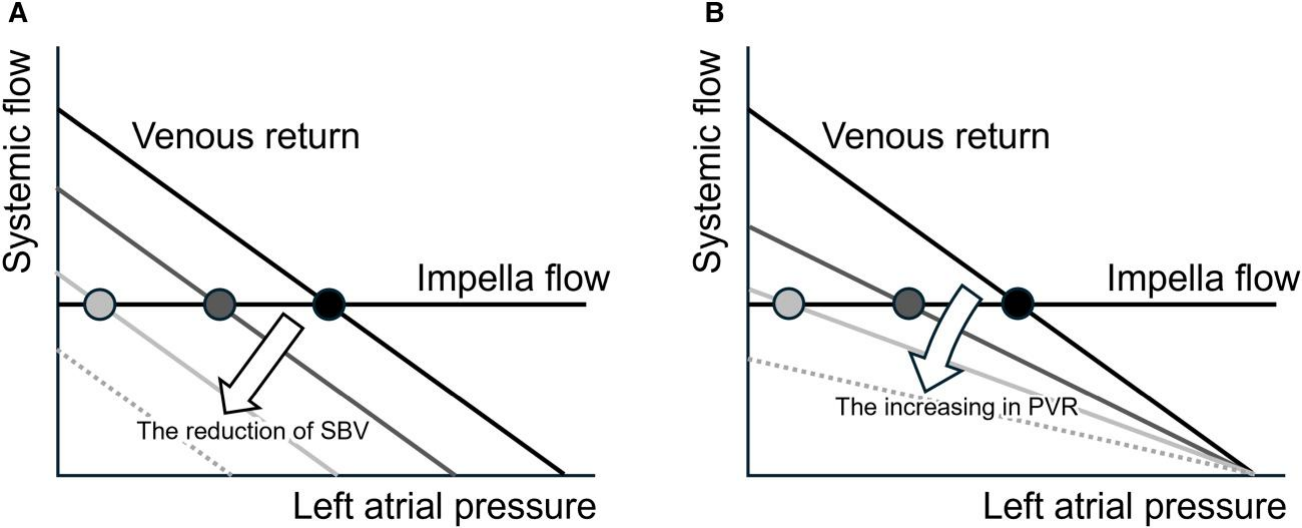
Schema of circulatory equilibrium during VF with Impella support. The intersection of the Impella flow rate and the venous return curve determines the haemodynamic operating point. The reduction in SBV shifts the venous return curve downward in parallel and moves the operating point to the left (*A*). An increase in the PVR decreased the slope of the venous return curve and moved the operating point to the left (*B*). SBV, stressed blood volume; PVR, pulmonary vascular resistance.

As shown in *Figure 2-A*, a stepwise decrease in the SBV eventually resulted in an LVP below 0 and failure of the Impella support. Because LV filling is a prerequisite for maintaining the Impella operation, it is possible to demonstrate the hypothetical shift of the operating point shown in *Figure 7-A*. In our study, gradual changes in SBV also reduced PAF and AP despite the presence of Impella support. This phenomenon can be explained by changes in the pressure gradient between (pump outlets) AP and LVP (pump outlet). Because Impella is an axial-flow pump, the greater the pressure difference between the AP and LVP, the lower the flow rate at a constant speed (*P* level). Our study demonstrated the importance of the SBV in the Impella during VF. Imamura *et al*.^11^ reported that a decrease in central venous pressure, a major component of SBV, due to diuretics resulted in low-output syndrome refractory to incremental rotational speed in the Fontan circulation and an LV assist device with RV failure circulation. Our results are consistent with these findings.

As shown in *Figure 7-B*, we also confirmed that high PVR limited LV filling and disturbed the stable operation of the Impella. This effect can also be explained by Guyton circulatory equilibrium, as shown in *Figure 6-B*. A simulation study conducted by Matsushita *et al*.^12^ found that, under conditions of severe RV dysfunction and high PVR, an increase in the Impella flow rate increased the RAP and decreased the LAP excessively, leading to LV suction and limiting the increase in total systemic flow. Hosein *et al*.^13^ reported that early and late mortality after the Fontan procedure correlated with elevated preoperative pulmonary artery pressures. They indicated that a high PVR complicated the management of patients who had undergone the Fontan procedure. Additionally, Imamura *et al*.^14^ reported that in patients implanted with an LV assist device, right ventricular dysfunction combined with PVR > 3.7 was a factor necessitating the use of an RV assist device.

Taken together, our observations and those of previous reports indicate that appropriate SBV and PVR are important for obtaining the operating point during VF with Impella support. In addition, our proposed theory, which is based on the circulatory equilibrium framework, can explain the haemodynamics in several case reports of VF with Impella support.

### Impact of Impella on central and peripheral perfusion

The present report is the first to demonstrate how the Impella support maintains systemic, cerebral, and coronary blood flow during VF. As shown in *Figure 5*, the Impella generated forward flow by increasing the PAP–LAP gradient, which had collapsed during VF, in a support-level–dependent manner. The maximum support level (P8) of Impella maintained 55.7 ± 20.8% of PAF, 72.5 ± 5.6% of CAF, and 68.8 ± 17.4% of CoF compared to the values at baseline in our study (*Figure 6*). Caminiti *et al*.^6^ reported a case in which Impella support preserved a 64-year-old man's blood pressure and consciousness during VF after primary PCI, and a single 200 J DC shock restored the sinus rhythm without removing the Impella. Our study is similar to their report in that the Impella was already in place before the onset of VF, and the haemodynamic mechanisms demonstrated in our model may help explain how blood pressure was partially preserved in their case despite the occurrence of VF.

Our study demonstrates that Impella maintains CAF, which is directly related to cerebral perfusion.^15^ Several studies have suggested that resuscitation with Impella is associated with improved neurological outcomes. Vase *et al*.^5^ reported that Impella alone restored circulation in patients with refractory cardiopulmonary arrest, leading to a 50% survival rate and favourable neurological outcomes, consistent with most studies on the effects of veno-arterial (V-A) ECMO in these patients. Another multicentre registry reported a 30-day survival rate of 37.1% without neurological impairment following Impella CP insertion during ongoing CPR.^16^ These findings suggest that Impella use during VF may improve neurological outcomes, which may be attributed to its ability to maintain the cerebral blood flow, as demonstrated in our study.

Finally, Impella support increased CPP, and P8 support achieved a CoF of 68.8 ± 17.4% (57.8 ± 20.6 mL/min) relative to baseline conditions. Previous studies established a strong correlation between CPP and CoF during CPR in experimental models.^17^ Additionally, both CPP and CoF have been linked to successful resuscitation. Paradis *et al*.^18^ reported a positive association between CPP and patient survival. In a porcine model of cardiac arrest, the use of Impella doubled the rate of return of spontaneous circulation and improved cardiac function in the early post-arrest period.^19^ In real-world scenarios of refractory VF, the deployment of Impella is logistically challenging. Ongoing CPR, profound haemodynamic collapse, and technical difficulties in establishing large-bore arterial access substantially limit the feasibility of rapid Impella implantation in such settings. Furthermore, all experiments were performed in animals with structurally normal coronary arteries. Thus, the preservation of coronary perfusion under Impella support demonstrated in our model cannot be extrapolated to conditions involving obstructed or partially obstructed coronary arteries, such as AMI or PCI-related VF. In these clinical scenarios, coronary flow may be critically impaired despite adequate Impella unloading, and the extent to which Impella support alone can maintain myocardial perfusion remains uncertain. This distinction is essential when interpreting the translational implications of our findings.

Importantly, the partial preservation of systemic, carotid, and coronary flows observed in this study does not indicate that Impella support alone is an adequate therapy during VF. In clinical practice, the manufacturer’s Impella manual clearly states that standard, guideline-directed CPR and defibrillation, not the Impella flow, constitute the primary and essential therapies for VF.^4^ The favourable haemodynamics in our model were achieved only under tightly controlled laboratory conditions: adequate stressed blood volume, relatively normal pulmonary vascular resistance, active preload optimization, and prior Impella insertion in fully anaesthetized and mechanically ventilated animals. These conditions differ fundamentally from the profound circulatory, respiratory, and metabolic disturbances typically encountered during clinical VF or cardiac arrest. Accordingly, our findings should be interpreted as physiological insights into the interaction between Impella support setting and haemodynamics during VF, rather than as evidence supporting any modification of clinical VF management or Impella deployment strategies.

## Limitation

Our study had several limitations. First, this study was exploratory in nature, and no prespecified effect sizes or hypothesis-testing framework were applied. In addition, the study was conducted as a small-scale animal experiment in goats under constrained experimental conditions. Protocol 1 used only one animal per haemodynamic manipulation, and Protocol 2 employed a fixed P0→P4→P8 sequence with a shortened P0 epoch and preload tuning using saline to maintain RAP and LVP above 10 mmHg. These factors may introduce within-animal biases and limit strong causal inferences. Therefore, the findings should be interpreted as concept-generating rather than providing definitive mechanistic evidence.

Second, there are several aspects of this study that differ substantially from clinical conditions. The absence of the distal tip may make the inlet position less stable, potentially affecting the timing or occurrence of suction events. This factor may have contributed to the suction timing observed in Protocol 1. In contrast, LV suction was not detected in Protocol 2, likely because preload tuning was performed to maintain adequate LV filling. Therefore, we consider that this issue does not affect the primary physiological conclusions of the manuscript. Respiratory conditions in this study may have favoured the maintenance of Impella-generated perfusion during VF. Pulmonary blood flow is determined by the PAP–LAP gradient, which is highly sensitive to ventilatory parameters, such as PEEP, intrathoracic pressure, and oxygenation. The animals were ventilated with normal lungs and a low PEEP of 3–5 cmH₂O under open-chest conditions, keeping intrathoracic pressure near atmospheric levels and likely maintaining PVR within a relatively low range. In clinical practice, however, higher PEEP is frequently required to manage pulmonary congestion, which can increase PVR,^20,21^ impede venous return, and heighten the risk of Impella-related LV suction.^12^ Hypoxia, which commonly accompanies elevated left atrial pressure in VF, also increases PVR^22^ and may further compromise left-sided perfusion. Therefore, the respiratory environment in our experiments may not fully reflect clinical conditions. Thus, these differences should be considered when interpreting the clinical relevance of our findings.

Third, the clinical significance of preserving arterial flows remains unclear. Although we measured carotid and coronary arterial flows as surrogate markers of organ perfusion, these indices do not directly reflect tissue-level perfusion or functional outcomes. Therefore, to extrapolate the flow-preserving effects of Impella support during VF to clinical settings, future studies should include evaluations of peripheral tissue perfusion and functional neurological or myocardial outcomes. In addition, although we used the Impella CP in this study, the results might have been significantly different if a higher-flow Impella (Impella 5.5®) device had been used.

Fourth, we assessed only acute haemodynamic responses over short periods of VF. Whether Impella support can sustain adequate circulation over longer durations, such as those encountered during PCI or prolonged cardiac arrest, remains unknown. In addition, maintaining the high preload required for optimal Impella performance during VF may be challenging in real-world clinical settings. For these reasons, caution is warranted when directly linking these findings to Impella-supported management of VF in clinical practice.

Fifth, although the experiments in Protocol 2 were conducted using the same P0, P4, and P8 sequences across all subjects, randomizing the sequence might have allowed for a clearer and more valid assessment of the effects of pressure and flow in each group.

## Conclusion

Under tightly controlled experimental conditions with adequate SBV and normal PVR, Impella support partially preserved systemic, carotid, and coronary perfusion in a goat model of VF. These findings offer physiological insights into the interaction between Impella support and haemodynamics during VF. However, confirmation of any clinical relevance will require further investigation in more clinically representative settings.

## Supplementary Material

oeaf173_Supplementary_Data

## Data Availability

The datasets generated and/or analysed during the current study are available from the corresponding author on reasonable request.
