# Supplementary material for: Impella preserves haemodynamics with adequate stressed blood volume and normal pulmonary vascular resistance in a goat model of ventricular fibrillation
Source: Eur Heart J Open. 2025 Dec 18;6(1):oeaf173. doi: 10.1093/ehjopen/oeaf173 (PMC12940114; doi:10.1093/ehjopen/oeaf173)
Supplement: oeaf173_Supplementary_Data [file oeaf173_supplementary_data.zip › supplemental_material_1.docx]

**Supplementary material 1**

**Electrocardiogram and Pressure Tracings Immediately After VF Induction in Goats (Protocol 2)**

Each figure presents snapshots of the monitoring screen showing the electrocardiogram (ECG) and haemodynamic pressure tracings immediately after ventricular fibrillation (VF) induction in all goats used in Protocol 2. The ECG clearly demonstrates VF in all animals. However, atrial contraction waves are detectable in goats just after the VF induction. These atrial contractions gradually disappeared as VF progressed, except for two goats in Protocol 2.

**#1**

**
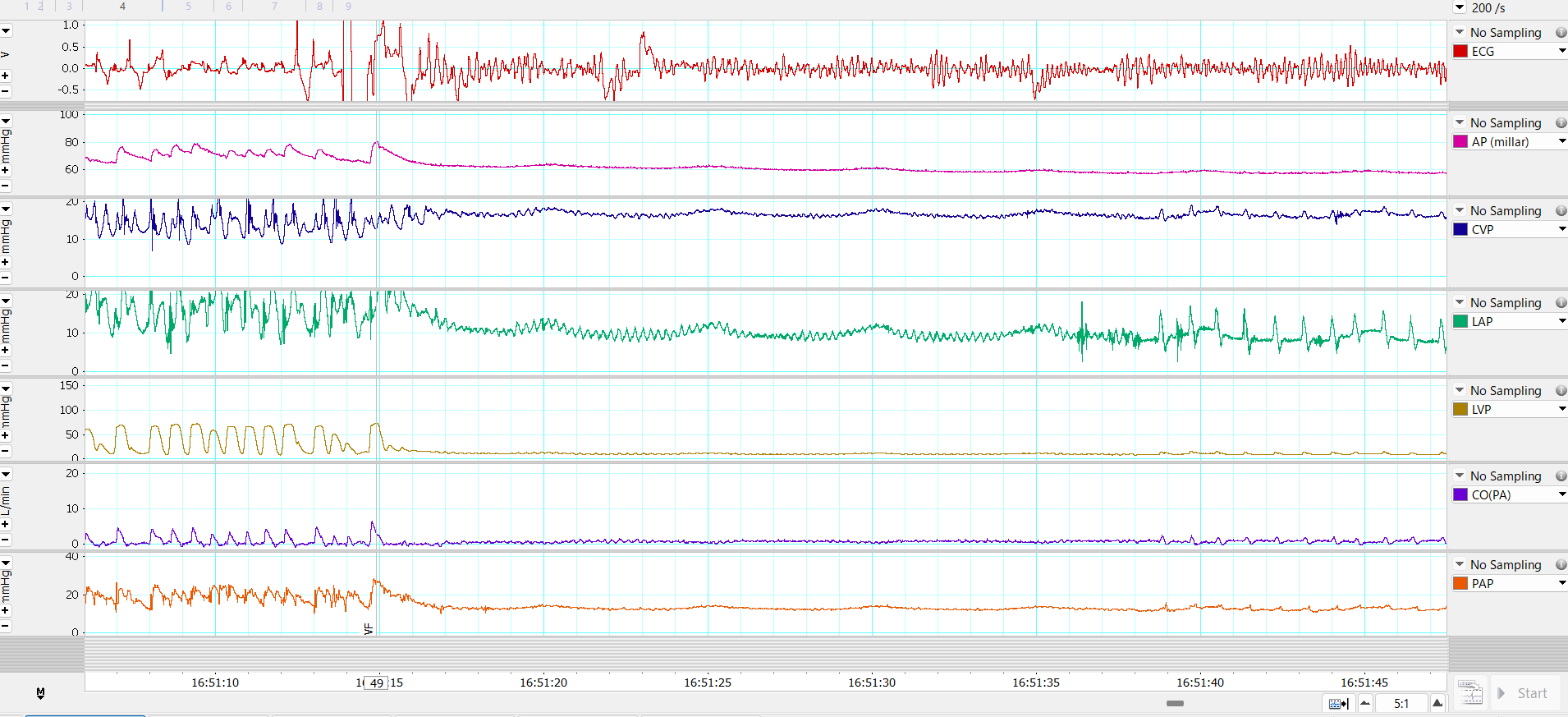
**

**#2**

**
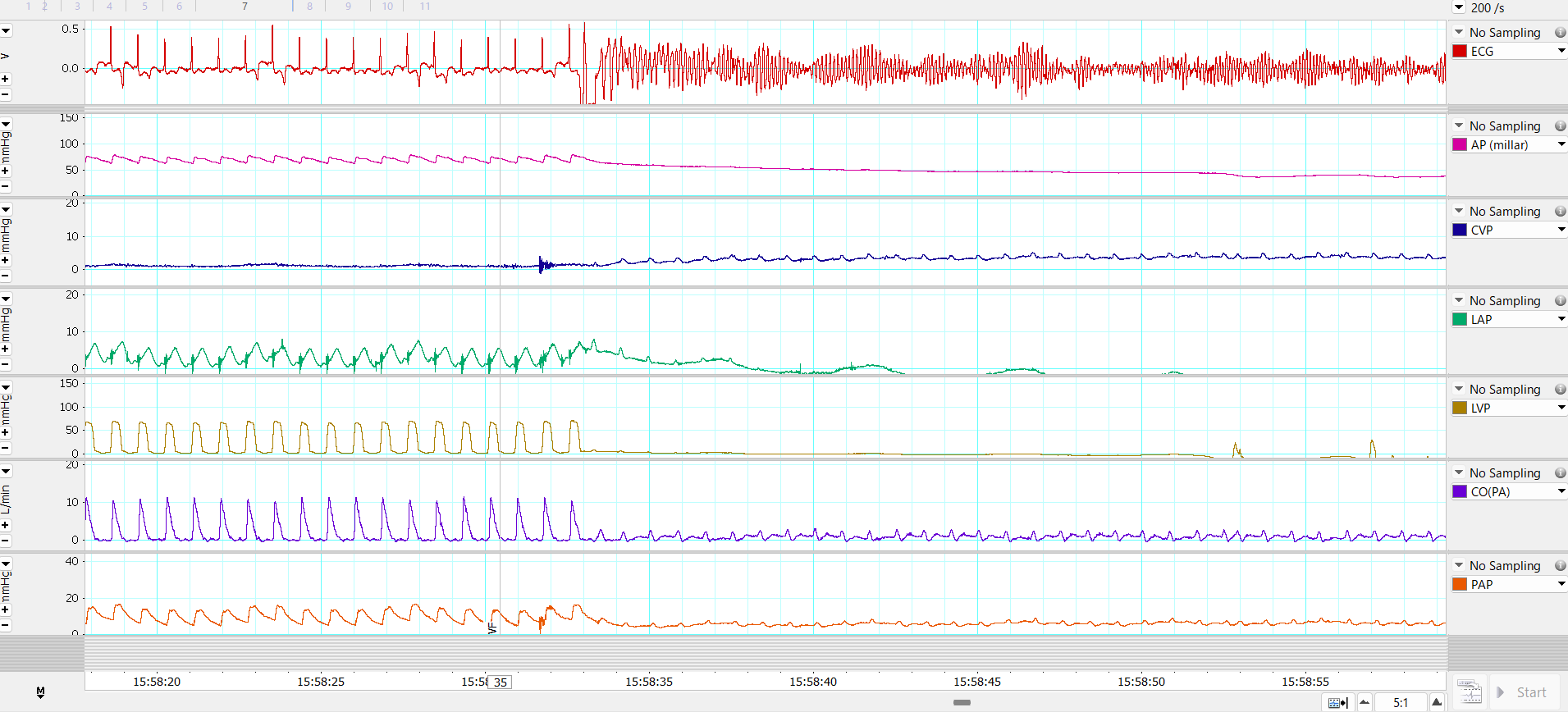
**

**#3**

**
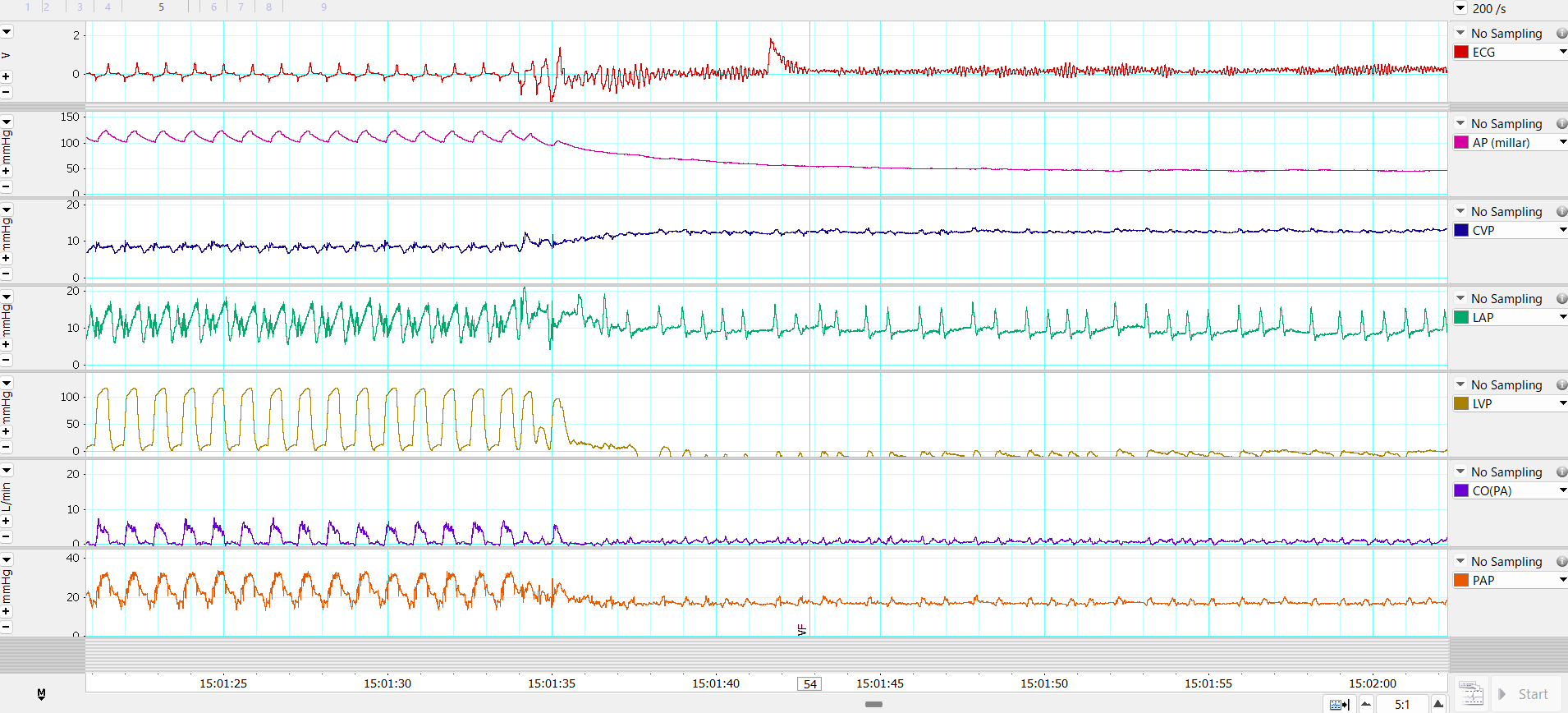
**

**#4**

**
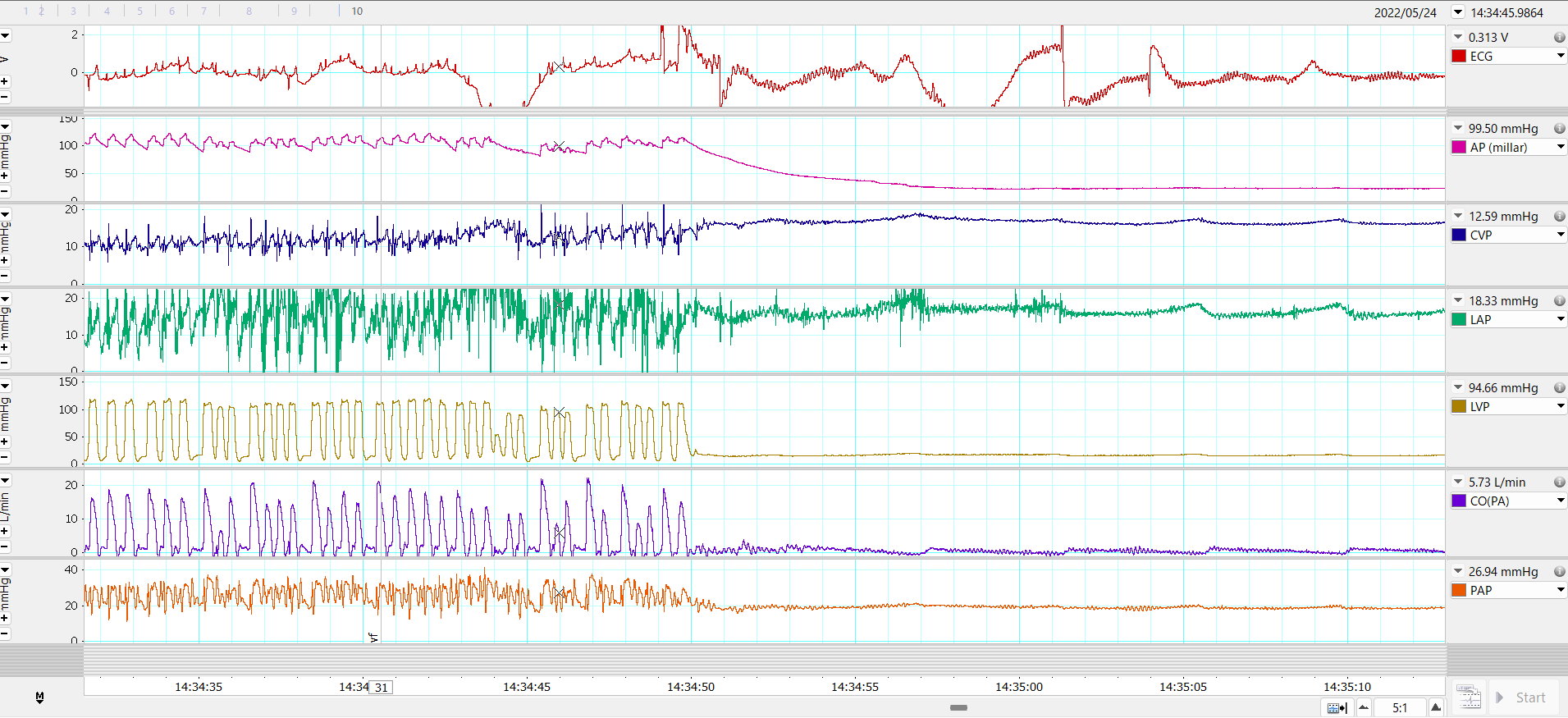
**

**#5**

**
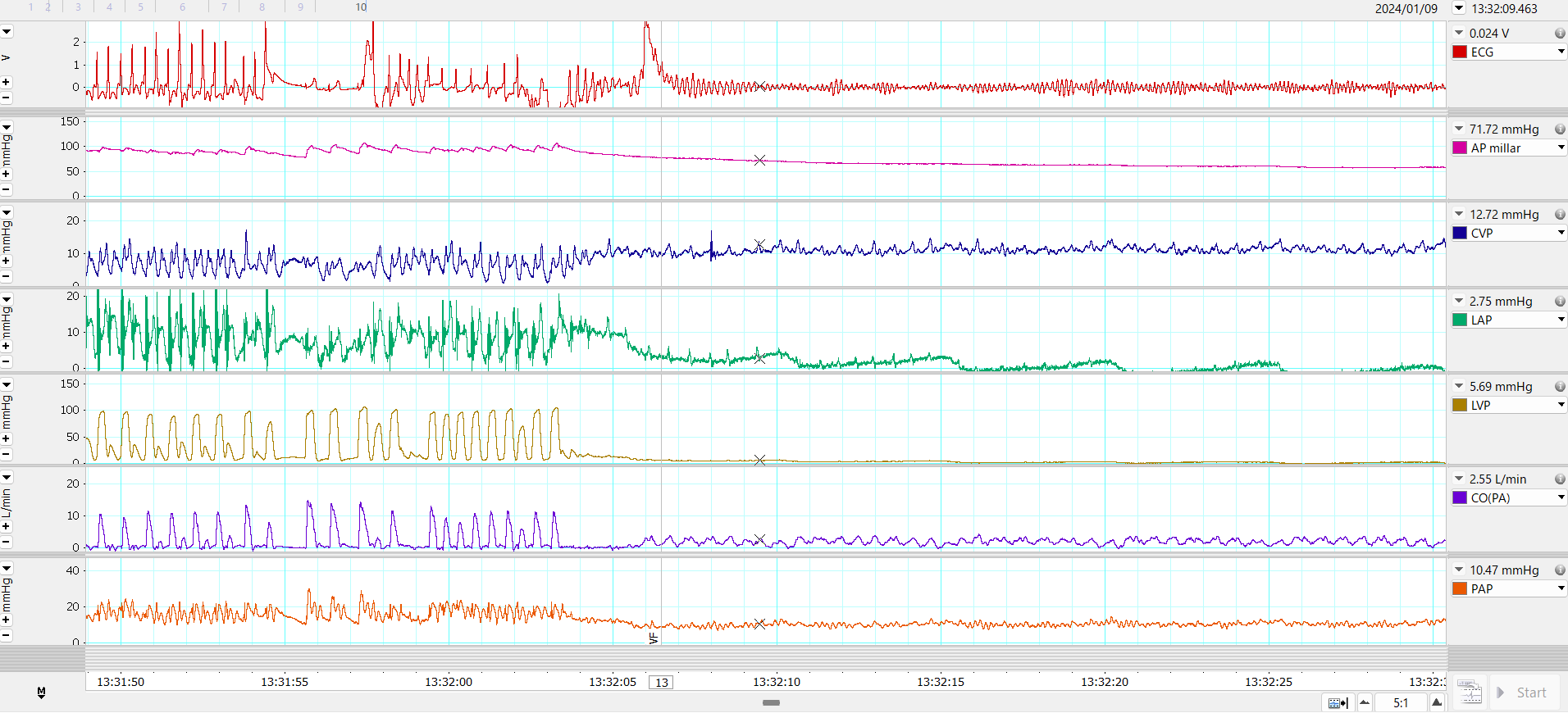
**

**#6**

**
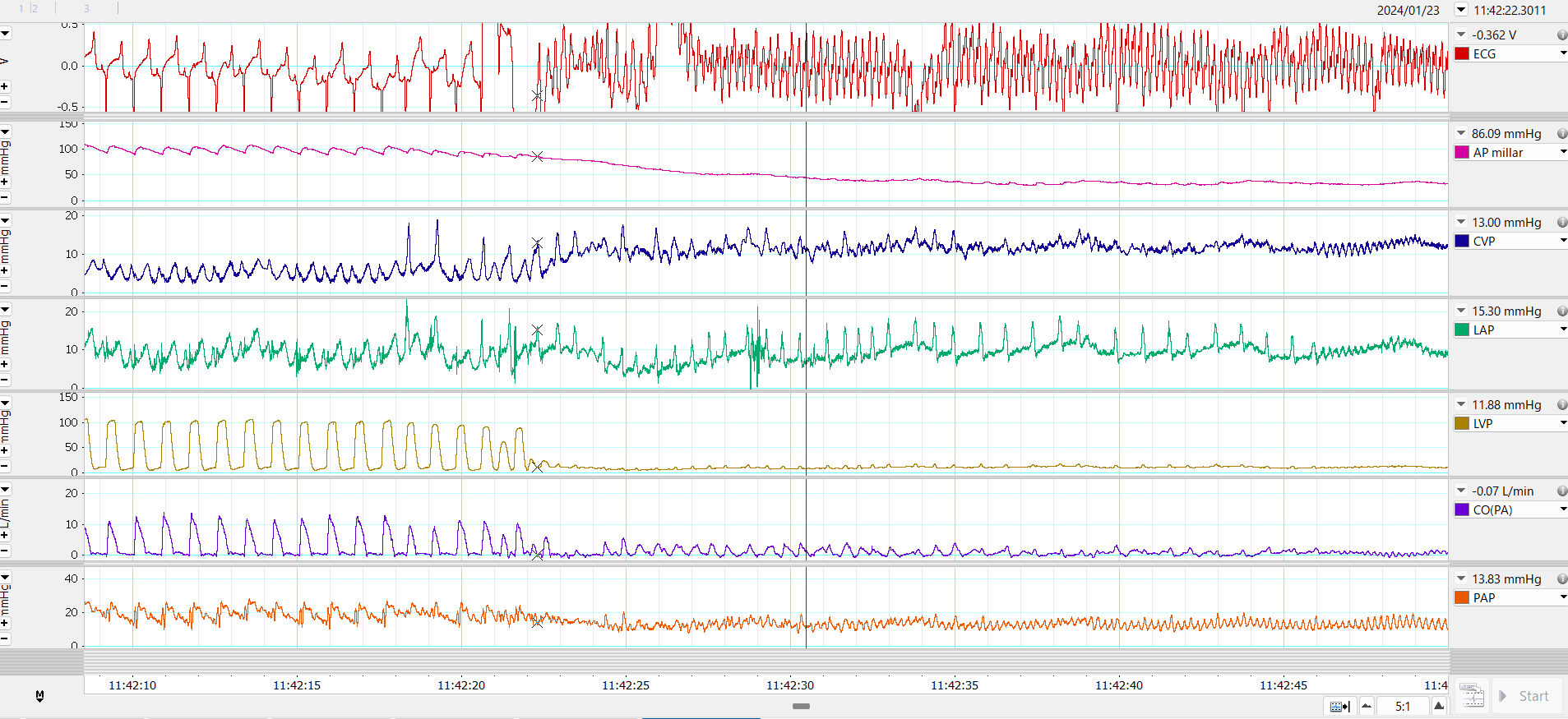
**

Although the monitor displays CVP, we directly recorded right atrial pressure (RAP) using the method described in the Methods section. ECG, electrocardiogram; AP, arterial pressure; CVP, central venous pressure; LAP, left atrial pressure; LVP, left ventricular pressure; CO, cardiac output; PA, pulmonary artery flow; PAP, pulmonary artery pressure.
