# Supplementary material for: Impella preserves haemodynamics with adequate stressed blood volume and normal pulmonary vascular resistance in a goat model of ventricular fibrillation
Source: Eur Heart J Open. 2025 Dec 18;6(1):oeaf173. doi: 10.1093/ehjopen/oeaf173 (PMC12940114; doi:10.1093/ehjopen/oeaf173)
Supplement: oeaf173_Supplementary_Data [file oeaf173_supplementary_data.zip › supplemental_material_2.docx]

**Supplementary material 2**

**Theoretical consideration of ventricular fibrillation haemodynamics under Impella support**

Guyton’s circulatory equilibrium, which consists of cardiac output (CO) and venous return (VR) curves, is a powerful framework for understanding circulatory physiology [1]. Sunagawa et al. [2] proposed an extended Guyton model that represents the cardiac pumping ability of the integrated CO curve and the VR function using the VR surface. In this framework, the VR for a given stressed blood volume (SBV) was calculated as a function of right (RAP) and left atrial pressures (LAP) as follows:

$VR=\frac{SBV}{W}-G_{P} \cdot LAP-G_{S}\cdot RAP$ …1

where W, G_P_, and G_S_ are constants.

Under ventricular fibrillation (VF) conditions, the right heart cardiac output (CO_R_) is expressed by the following equation in accordance with Ohm's law:

$CO_{R}=\frac{RAP-LAP}{PVR}$　　　　　…2

From Equations 1 and 2, the relationship between VR and LAP can be expressed as follows:

$VR=\frac{SBV}{\left( 1+G_{S}\cdot PVR \right)W}-\frac{G_{S}+G_{P}}{1+G_{S}\cdot PVR}LAP$ …3

This equation represents the effects of SBV and PVR on the VR of the LV during VF.

Under VF conditions with Impella support, the left heart cardiac output (CO_L_) was determined using the Impella flow. Thus, the CO_L_ curve can be expressed as

$CO_{L}=Impella flow$ …4

Considering this theory and based on the circulatory equilibrium framework, we hypothesised that Impella operation during VF requires adequate SBV and low PVR. We proved this hypothesis using Protocol 1.

1. Guyton AC, Coleman TG, Jones CE. Circulatory physiology: Cardiac output and its regulation. 2nd ed. Philadelphia, PA: Saunders;1973. p237–252.
2. Sunagawa K, Sagawa K, Maughan WL. Ventricular interaction with loading system. Ann Biomed Eng 1984;12:163–189.
